# BeitAI-pHLA: Multiallele Peptide–HLA Class I Binding Prediction Using Protein Language Model and Multi-Instance Learning

**DOI:** 10.34133/csbj.0220

**Published:** 2026-09-11

**Authors:** Shigang Qiu, Yong Sun, Xiaofei Ye

**Affiliations:** ^1^ Kindstar Biotech, Wuhan 430000, China.; ^2^ Kindstar Global Precision Medicine Institute, Wuhan 430000, China.; ^3^ Department of Rheumatology and Clinical Immunology, Peking University First Hospital, Beijing, China.

## Abstract

Human leukocyte antigen (HLA) molecules participate in cellular immune responses by binding to peptide fragments derived from antigens. Exploring this process is crucial to understanding the mechanisms and underlying factors that regulate the cellular immune system. Due to the extreme polymorphism of HLA, the peptides obtained from mass spectrometry of eluted ligand experiments typically correspond to multiple HLA alleles. They are thus poly-specific, providing various options for HLA assignments. This introduces notable challenges for accurate peptide–HLA assignment and interpretation. To address this limitation, we present BeitAI-pHLA, a deep learning framework for predicting peptide–HLA class I binding by integrating protein language model embeddings with an attention-based multi-instance learning mechanism. Our evaluations on benchmark and external datasets indicate that BeitAI-pHLA markedly outperforms other advanced prediction tools across multiple external validation datasets, achieving an area under the precision-recall curve of 0.967 on the multiallele dataset and maintaining robust performance under varying negative peptide proportions. Furthermore, BeitAI-pHLA demonstrates superior motif deconvolution capability, with higher motif similarity (13.6% increase in position-specific scoring matrix correlation coefficient), and achieves the best area under the precision-recall curve in immunogenic neoepitope prediction, offering improved prioritization of candidate peptides for immunogenicity evaluation. BeitAI-pHLA provides a highly accurate tool for binding prediction and deconvolution in multiallele immunopeptidomics data, offering considerable potential for biomedical research and clinical translation. The BeitAI-pHLA prediction tool is accessible at https://phla.kindstarbiotech.com/, and the source code is available at https://github.com/KindstarGlobalInstitute/BeitAI-pHLA.

## Highlights

• Novel integration of a protein language model with a capsule network provides a deep and context-aware representation of peptide–HLA interactions.
• An attention-based multi-instance learning mechanism directly addresses the challenge of allele deconvolution in multiallele immunopeptidomics data.
• Demonstrates superior accuracy and robustness, achieving an AUPRC of 0.967 on multiallele data and maintaining performance under high decoy peptide ratios.
• Achieves higher motif similarity (13.6% improvement in PSSM correlation) and the best AUPRC for immunogenic neoepitope prediction among peer tools.

## Graphical Abstract

**Figure:**
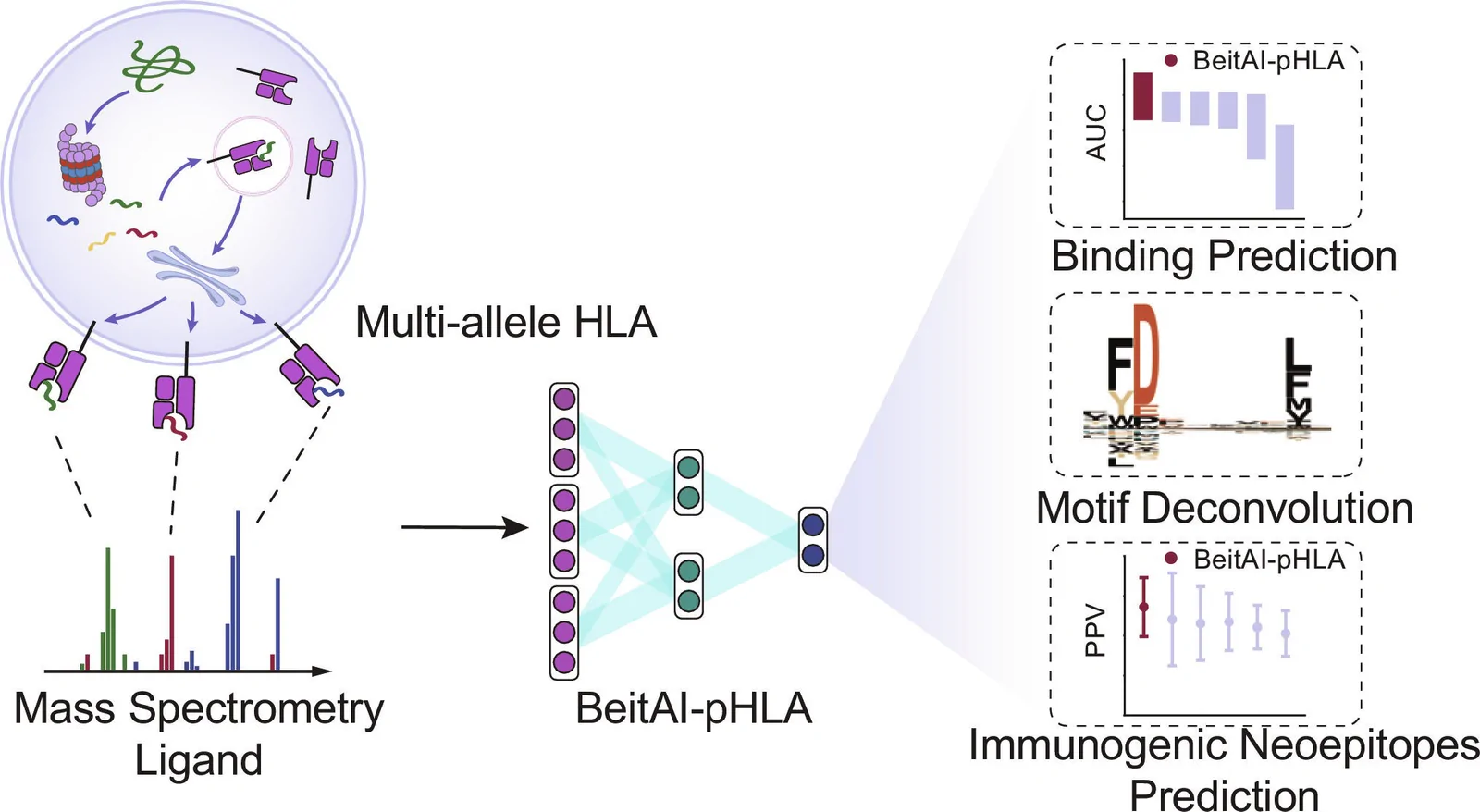

## Introduction

Human leukocyte antigen (HLA) molecules are essential cell surface proteins involved in antigen presentation and are expressed on virtually all nucleated cells. Peptides generated from the proteasomal degradation of intracellular proteins bind to HLA molecules to form peptide–HLA (pHLA) complexes that are displayed on the cell surface [1]. T cells can specifically recognize and respond to antigens presented by HLA molecules, particularly those derived from cancer cells or virus-infected cells, thereby triggering an appropriate immune response. In adaptive immunity, antigen presentation is a prerequisite for inducing cytotoxic T-cell responses [2,3]. Thus, the binding of antigenic peptides to HLA molecules is a critical step in cellular immunity. Understanding this process has substantial implications for cancer immunotherapy and vaccine design [4–6].

HLA molecules comprise 2 isotypes that present different antigenic peptides. HLA class I molecules present endogenous antigens derived from intracellular protein degradation, with typical peptide lengths of 8 to 14 amino acids [7]. In contrast, HLA class II molecules present exogenous antigens derived from extracellular proteins, with peptides generally 12 to 25 amino acids [8]. HLA class I molecules are encoded by 3 common genes (*HLA-A*, *HLA-B*, and *HLA-C*). These loci exhibit extensive polymorphism, with more than 27,000 HLA-I alleles recorded in the HLA database [9]. Different HLA-I alleles have distinct peptide-binding specificities, identifying which peptides bind to each allele within the enormous space of potential antigens remains a major computational and experimental challenge.

In recent years, the rapid accumulation of in vitro affinity data and mass spectrometry (MS)-based immunopeptidomics datasets has enabled substantial advances in deep learning-based pHLA class I binding prediction. These methods commonly formulate peptide–HLA binding as a text classification problem, training a deep learning model directly on amino acid sequence data [10–13]. Compared with in vitro affinity data, MS peptidomics datasets directly measure pHLA complexes, providing comprehensive results of antigen processing and presentation. However, the data generated in MS experiments are inherently ambiguous: Each human cell can express up to 6 HLA-I alleles, such that the immunopeptidome acquired by MS is a mixture of all ligands presented by these alleles [14]. This multiallele (MA) nature complicates both ligand assignment and downstream model training, presenting a major challenge for accurate pHLA binding prediction using MS data.

In published studies on pHLA binding affinity prediction, the latest versions of NetMHCpan (NetMHCpan4.1) [12] and MHCflurry (MHCflurry2.0) [13] are considered state-of-the-art and most commonly used predictors for HLA class I peptide binding. MHCflurry is trained on MS single-allele (SA) data and affinity measurements, combining binding models and antigen-processing models for prediction, rather than directly incorporating MA data into model training. NetMHCpan employs a semisupervised learning approach [15] to annotate MA data to SA data in the training set, utilizing a shallow feedforward neural network with a single hidden layer. Additionally, both predictors use amino acid embedding methods based on the Blocks Substitution Matrix (BLOSUM) matrix [16] and sparse encoding to characterize the input peptide and HLA sequences, yielding lower-dimensional representations. More recently, TripHLApan [17] introduced a transfer-learning framework incorporating gated recurrent units to build allele-specific binding models, further improving SA prediction performance. Despite these advances, most predicted neoantigen candidates lack immunogenicity [18–20]. Thus, substantial opportunities remain to improve the accuracy and biological relevance of (neo)antigen prediction, particularly for MA eluted ligand datasets, which constitute a large and practically important portion of immunopeptidomics data.

To overcome this limitation, we propose a deep learning model, BeitAI-pHLA (named after a pHLA Binding Estimation using Immune Technology of Artificial Intelligence), to predict pHLA class I binding. BeitAI-pHLA introduces 3 key innovations: (a) Replacing traditional BLOSUM matrix-based encoding with a protein language model (evolutionary scale modeling [ESM]) for embedding peptide and HLA sequences, thereby capturing richer, context-aware amino acid representations; (b) Integrating a capsule neural network architecture that empirically captures hierarchical feature interactions and relative positional relationships critical for pHLA binding; (c) Implementing an attention-based multi-instance learning (MIL) mechanism [21], where each sample is a bag of HLA alleles and the model learns which allele each peptide binds to, to address the ambiguity inherent in MA data. Unlike semisupervised methods such as NetMHCpan4.1, BeitAI-pHLA employs an end-to-end MIL framework trained directly on MA data, thereby avoiding the error propagation introduced by predecomposition. Trained and evaluated on large-scale MS immunopeptidomics datasets, BeitAI-pHLA consistently outperforms existing methods across multiple independent test settings.

## Materials and Methods

### Training data

We collected the HLA class I binding prediction dataset from IEDB (http://tools.iedb.org/main/datasets/) and filtered the dataset to exclude binding samples from nonhuman species, focusing specifically on HLA presentation prediction. The samples in this dataset fall into 2 types: binding affinity scores from in vitro affinity experiments and binding annotations derived from eluted ligand experiments. Binding affinity data represent only a single event of pHLA binding (neglecting other biological characteristics) of the antigen presentation pathway. Therefore, we elected to retain only MS-eluted ligand data to avoid biases that may arise from affinity-based measurements. All MS datasets included in the original studies had undergone standard quality-control procedures, typically involving false discovery rate filtering. To construct the training set, we removed all records with identical pHLA allele pairs to eliminate potential redundancy. However, instances in which the same peptide sequence appeared with different HLA alleles were preserved, as these reflect authentic multispecific binding events.

Negative samples were generated by randomly sampling peptides from the human proteome (Swiss-Prot [22]). During sampling, we ensured that the length distribution of negative peptides exactly matched that of the positive peptides in the dataset. This strategy prevents the model from exploiting peptide length as a trivial discriminative feature and ensures that decoy peptides originate from a biologically realistic sequence background. Although random sampling of the proteome cannot fully reflect biological processes such as proteasomal cleavage or TAP (transporter associated with antigen processing) transport, this strategy has been widely adopted in the field (NetMHCpan, MHCflurry, MixMHCpred, etc.) due to the lack of experimentally validated nonbinders in public datasets.

Consequently, we obtained a pHLA binding dataset comprising 10.67 million MS-derived records spanning 144 distinct alleles. The dataset contains both SA and MA entries at an approximate ratio of 1:2, with a positive-to-negative (decoy) ratio of 1:20. To further prevent data leakage between the training and test sets at both the sequence level and the motif level, we implemented a 2-layer clustering strategy. First, we applied CD-HIT [23] with an 85% similarity threshold to group peptide sequences with high overall sequence identity. Second, we performed an anchor-aware clustering step to prevent motif-level leakage. For each peptide, we extracted 6 anchor residues: $P_{1}$, $P_{2}$, $P_{3}$, $P_{n-2}$, $P_{n-1}$, and the C terminus ($P_{n}$), and grouped peptides by their 6-anchor signature. We then merged any CD-HIT clusters sharing at least 1 identical anchor signature into a single supercluster. These superclusters were treated as the smallest indivisible units during data partitioning, ensuring that peptides with identical anchor patterns were strictly prohibited from appearing across different splits. Detailed descriptions of the test sets are provided in the “SA evaluation dataset” and “MA evaluation dataset” sections.

### SA evaluation dataset

Two datasets were used to validate the SA data performance. The first is an external SA validation set sourced from the MS ligand dataset on the NetMHCpan4.1 online platform, comprising 377,328 entries. The majority of HLA alleles in this dataset either overlap with those in our training set or belong to the same supertype. The second is an unseen-allele SA test set of 312,295 entries, constructed specifically to assess pan-allele generalization on HLA alleles completely absent from training. To ensure that these unseen alleles are genuinely novel in sequence space, we clustered all HLA alleles by edit distance on their 34-residue pseudo-sequences with a threshold of 5 and selected 9 alleles from the smallest clusters as the held-out set (details are reported in Table S1). All evaluation datasets were compared against our training data to remove redundant entries that shared identical ligand sequences and HLA molecules.

### MA evaluation dataset

In addition to the MA test dataset randomly selected from our training dataset, we also used immunopeptidomics data from 21 meningioma samples [24,25] as an external validation dataset to validate the performance of BeitAI-pHLA. The MA external dataset comprises 78,011 HLA-I-positive ligands and 312,044 decoy peptides (a ratio of 1:4). Notably, these data do not overlap with the training dataset.

### Immunogenic neoepitopes evaluation dataset

For predicted peptides, whether they elicit an immunogenic response is crucial for downstream tasks. Thus, we evaluated and compared the ability of various models to identify immunogenic peptides using the dataset described by Xu et al. [26]. This dataset includes 349 immunogenic neoepitopes and 1,838 nonimmunogenic neoepitopes.

### Evaluation metrics

The pHLA binding prediction problem is framed as a binary classification task in machine learning. We utilized several model evaluation metrics commonly used in machine learning classification tasks, which have been widely applied in previous pHLA binding predictors. The metrics used include:

1. AUROC (area under the receiver operating characteristic curve) is the area under the curve composed of sensitivity and specificity, commonly referred to as AUC. AUPRC (area under the precision-recall curve) is the area under the curve composed of precision and recall.

2. F1 score, a metric that considers both precision and recall, provides a balanced measure of a classifier’s performance.

$$F1\;\text{score}=\frac{2\times \text{Precision}\times \text{Recall}}{\text{Precision}+\text{Recall}}$$ (1)

where

$$\text{Precision}=\frac{\mathrm{TP}}{\mathrm{TP}+\mathrm{FP}}$$ (2)

$$\text{Recall}=\text{Sensitivity}=\frac{\mathrm{TP}}{\mathrm{TP}+\mathrm{FN}}$$ (3)

Another similar metric

$$\text{Specificity}=\frac{\mathrm{TN}}{\mathrm{TN}+\mathrm{FP}}$$ (4)

TP, FP, TN, and FN represent the number of true positives, false positives, true negatives, and false negatives, respectively.

3. Positive predictive value (PPV) indicates the proportion of samples predicted to be positive by the classifier that are actually positive, reflecting the accuracy of the classifier when predicting positives. The formula for PPV is the same as that for precision.

To ensure fair comparisons with existing tools that report %rank values, we normalized %rank scores to a 0 to 1 scale using the transformation score = 1 − (rank/100), aligning them with the probability outputs of binary classifiers.

### Sequence embedding of the predictor

Considering that HLA-binding peptides are typically 8 to 15 amino acids in length, BeitAI-pHLA is designed to support peptides of up to 15 amino acids. To standardize input peptide sequences to a length of 15 amino acids, we follow the method used in the ESM [27]. ESM is a transformer-based protein language model that can capture effective encodings and deep representations of protein sequences. For peptides shorter than 15 amino acids, special “<pad>” tokens are used to fill the remaining positions. The processing of HLA molecule sequences mirrors that of NetMHCpan, using a pseudo-sequence of fixed length (34 amino acids) that represents the core residues involved in binding rather than the entire sequence. Peptide sequences and HLA sequences are concatenated with a “-” symbol into a combined sequence, with “<cls>” and “<eos>” tags added at the beginning and end, respectively. After this processing, an input sequence of length 52 is obtained.

Specifically, we use version ESM2, a universal protein language model capable of predicting structure, function, and other protein properties directly from single sequences. Taking both performance and training time into consideration, we selected ESM2-150M as our ESM embedding model. To validate the use of the HLA pseudo-sequence (34 residues, representing the binding groove) as input, we also trained a variant using the full-length HLA sequence (360 to 365 residues, signal peptide removed) obtained from the IPD-IMGT/HLA database [9]. The performance difference between the pseudo-sequence and full-length inputs was negligible (AUPRC difference ≤ 0.0071 on both SA and MA test sets; Table S2), confirming that the pseudo-sequence does not compromise ESM2’s representation quality. During the training of the BeitAI-pHLA model, the ESM model’s parameters remain fixed and do not participate in parameter updates. After encoding by ESM2-150M, each token (amino acid or padding) is represented as a 640-dimensional vector, yielding a 52 × 640 2-dimensional input matrix. This matrix is then fed into the subsequent convolutional layers.

### Capsule neural network architecture

The neural network module consists of convolutional and capsule network layers. The convolutional layers use a multilayer structure inspired by residual networks to alleviate vanishing-gradient effects. Each convolutional block consists of 2 layers: a dimension control layer (kernel size 1) to align dimensions, and a feature extraction layer with 256 and 768 filters (kernel sizes 3 and 5, respectively). Both the beginning and end of the feature-extraction stack consist of 3 repeated 1×1 convolutions, each followed by batch normalization and rectified linear unit activation. In total, the convolutional module has 20 layers, with 2 residual connections, and no pooling is performed, preserving all features.

Although convolutional neural networks effectively capture local features, they are limited in modeling relative positional and directional relationships. Capsule networks address this limitation by replacing scalar neurons with vector-valued capsules that encode both the presence and geometric attributes of features. The primary capsule layer receives inputs from the convolutional layer and passes them to higher-level capsules through a transformation weight matrix.

The capsule layer produces 20 capsules, each a 10-dimensional vector, yielding a flattened 200-dimensional output. The number 20 was chosen to provide sufficient capacity to represent the diverse binding modes of HLA alleles in the training set, while remaining computationally feasible. Following the standard practice in capsule networks [28], each 10-dimensional capsule vector is used to simultaneously encode both the existence probability and the instantiation parameters (e.g., pose and deformation) of the learned features.

Details of the mathematical formulation of capsule network operations, including the dynamic routing process and the softmax function used to calculate attention weights, are provided in the Supplementary Materials.

### MIL architecture

Due to the multispecificity of MA data, elution MS labels overlap, making it difficult to determine the exact number of HLA molecules each peptide binds to. To address this ambiguity, we frame the problem as an MIL task, in which each HLA allele sequence is treated as an instance and the set of alleles expressed by a cell line or sample constitutes a bag. The instances are unlabeled, whereas each bag carries a binary label, defining a weakly supervised learning setting. A key requirement in MIL is capturing the relationship between instances and bags. We employ an attention mechanism that assigns higher weights to allele sequences that contribute more strongly to peptide binding. Specifically, the capsule network produces a 200-dimensional vector. A 3-head attention module then computes a 1 × 600 bag representation that integrates information across all alleles.

A fully connected block with 2 layers and sigmoid activation functions predicts the pHLA binding probability. For deconvolution, we average the 3 attention heads and assign each peptide to the HLA allele with the highest mean attention score, thereby identifying the most likely binding allele(s). The mathematical details of the attention-based MIL module are provided in the Supplementary Materials.

### Model training hyperparameters

All models were implemented using the PyTorch framework. To prevent overfitting, we performed 3 repeated random subsampling validations and dropout regularization with a rate of 0.3. The loss function was set to binary cross-entropy loss, and the model was optimized with the AdamW optimizer at a learning rate of 5 × 10^−5^ and a weight decay of 1 × 10^−5^. To compensate for the substantial class imbalance in the training data, we conducted a sensitivity analysis and assigned a weight of 20 to the positive class in the loss function. During training, we used a batch size of 128 and trained for a maximum of 20 epochs. Early stopping was applied with a patience of 3 epochs based on the validation loss, and the model checkpoint that achieved the best validation AUPRC was retained for final evaluation. The training set was randomly split into 80% for training and 20% for validation, with the validation split used solely for early stopping and hyperparameter selection. All reported results are from independent test sets that were not used during any stage of model development.

### Ablation variants for architecture comparison

To isolate the capsule network’s contribution, we constructed 2 ablation variants replacing capsule layers with a Transformer encoder (2 layers, 640 dim, 8 heads) or a multilayer perceptron (MLP; 2 fully connected layers, 1280 dim). All other settings were kept identical to those of BeitAI-pHLA.

### Motif visualization

In motif deconvolution, for each peptide in a MA sample, we compute the mean attention score across the 3 heads for each HLA allele. The peptide is then assigned to the allele with the highest mean attention score. To generate binding motifs for a given allele, we collect all peptides assigned to that allele and apply Seq2Logo [29] with default parameters (prior weight set to 0). Separate motifs are generated for each peptide length (8 to 14 amino acids). For single-allele data, the attention mechanism is bypassed, and all peptides are directly assigned to the sole HLA allele. In the logo plots, different colors represent distinct chemical properties of amino acids: negatively charged (red), positively charged (yellow), polar (cyan), or hydrophobic (black).

### Motif similarity

Position frequency matrix (PFM) and position-specific scoring matrix (PSSM), obtained from Seq2Logo, were used to represent the binding motifs for each allele. The PFM is constructed by calculating the frequency of each amino acid at every position within known binding peptides, reflecting a consensus sequence pattern. The PSSM extends the PFM by converting these frequencies into log-likelihood scores and incorporating background amino acid frequencies. This transformation highlights residues enriched beyond random expectation and provides a more sensitive measure of biologically meaningful binding preferences. Both the PFM and PSSM are *n* × 20 matrices, where *n* denotes peptide length. To quantify motif similarity, we computed Pearson correlation coefficients (PCCs) between corresponding matrices for each allele across methods.

## Results

### BeitAI-pHLA model overview

**Fig. 1.**
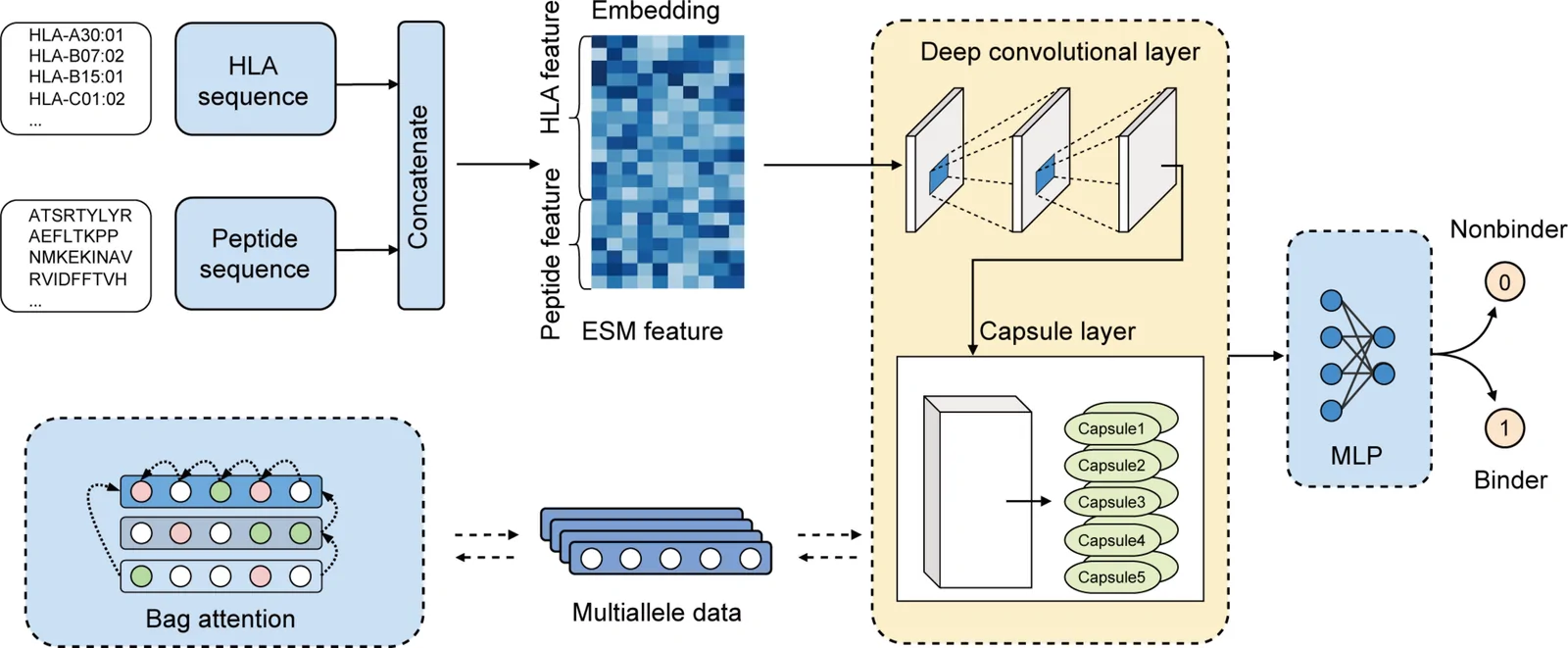
Building blocks of BeitAI-pHLA. BeitAI-pHLA comprises 3 parts: data embedding, feature extraction, and multiallele (MA) deconvolution. First, the combined human leukocyte antigen (HLA) sequence and peptide sequence were passed through the protein language model to obtain amino acid embedding vectors. The single-allele (SA) data embeddings are then directly fed into the feature extraction module, which contains capsule neural network blocks. The MA data were annotated as SA data using a multi-instance learning mechanism before entering the capsule network blocks, thereby covering all HLAs presented. Finally, the extracted features are fed into fully connected layers for the final classification.

To evaluate the probability of peptide presentation by HLA-I molecules, we developed BeitAI-pHLA, which was trained on eluted ligand MS data (Fig. 1). Specifically, we employed a protein language model called ESM for protein sequence embedding. ESM is a protein sequence language model with a Transformer architecture that can generate feature representations from a single protein sequence [27]. To enhance the learning of potential features and sequence relationships, we integrated both deep neural network and capsule neural network structures [28], which have been used in computational biology tasks [30–33]. For handling MA data, we adopted an attention-based MIL framework [34], which assigns allele-level scores and identifies the most likely binding allele for each peptide. The MIL parameters are updated at every training step to ensure sufficient learning epochs, unlike the semisupervised learning approach mentioned above. This model outputs a score indicating the likelihood that the input peptide binds to the HLA molecule. If the HLA is MA, the model can output the most probable HLA allele assignment.

### Reliable performance on SA data

**Fig. 2.**
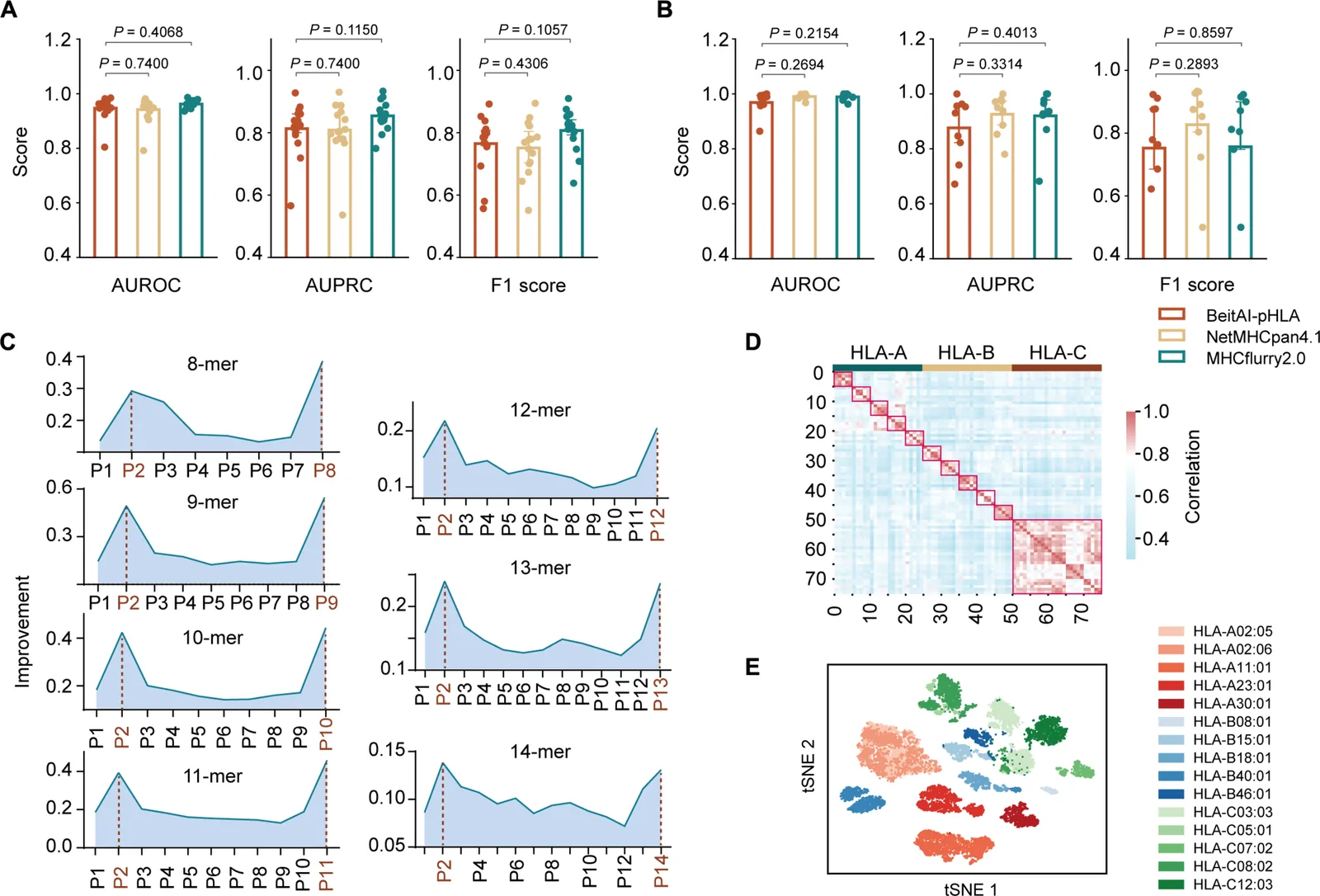
BeitAI-pHLA single-allele (SA) prediction performance and anchor position analysis. Area under the receiver operating characteristic curve (AUROC), area under the precision-recall curve (AUPRC), and F1 score performance metrics on the SA external dataset (A) and unseen allele dataset (B). (C) Single-position mutations characterize the contribution of different positions to the interaction between peptides and human leukocyte antigen (HLA) molecules, with peptide lengths ranging from 8 to 14 amino acids. (D) Correlation of binding characteristics for *HLA-A*, *HLA-B*, and *HLA-C*. Five alleles were selected for each of *HLA-A*, *HLA-B*, and *HLA-C*, and the binding feature representations were extracted for each allele with 5 randomly selected positive peptides. The Pearson correlation coefficient (PCC) was then calculated between the feature representations. (E) t-Distributed stochastic neighbor embedding (tSNE) projection of latent features from the final fully connected layer. Each point represents a peptide–HLA pair (single-allele data), colored by HLA family: red for *HLA-A*, blue for *HLA-B*, and green for *HLA-C*.

To evaluate the performance of BeitAI-pHLA in predicting pHLA affinity under SA scenarios, we tested it on the external SA datasets. Figure 2A shows that BeitAI-pHLA achieved comparable performance to that of NetMHCpan4.1 and MHCflurry2.0 across several metrics (Table S3). To assess generalization to unseen HLA alleles, we constructed a stringent HLA-cluster holdout set based on pseudo-sequence edit distance clustering (see the “SA evaluation dataset” section). On these held-out alleles, BeitAI-pHLA achieved an average AUROC of 0.969 and AUPRC of 0.876 (Fig. 2B and Table S3), with no marked performance drop compared to the standard split. These results indicate that our model performs comparably to state-of-the-art methods such as NetMHCpan and MHCflurry and can effectively generalize to novel alleles.

Anchor positions are crucial for peptide binding to HLA molecules. Some positions within the peptide sequence serve as anchors for HLA binding, while others are exposed for T cell receptor (TCR) recognition. Previous studies have shown that the second and final positions of epitope peptides typically act as residues for binding to HLA [35,36]. Because our attention mechanism operates at the allele level rather than the residue level, we performed a systematic single-position mutation scan to identify the peptide positions critical for binding prediction. Based on the positive data in the evaluation dataset, peptides of lengths 8 to 14 were selected, with an equal number per length interval. We then calculated the predicted scores for all possible amino acids at each position in these peptides and compared them with those of the original peptide sequences. This approach allows us to observe the impact of changes at key positions on binding affinity. The results in Fig. 2C show that alterations in the amino acids at the second and final positions of peptides of different lengths have the most pronounced impact on the predicted binding scores. Using the same approach, we further analyzed the classical influenza epitope GILGFVFTL (*HLA-A02:01*-restricted), and the resulting amino acid importance heatmap (Fig. S1) shows that substitutions at *P*_2_ (I), *P*_4_ (G), and *P*_9_ (L) most strongly reduce the predicted binding score. These results align well with established biological knowledge and provide first-order interpretability of the model’s decision-making process.

Subsequently, we extracted the feature vectors processed by the neural network method and calculated their PCCs to explore the model’s feature-extraction patterns for interactions. Figure 2D shows the visualization results of binding features for *HLA-A*, *HLA-B*, and *HLA-C* across different sequences, indicating that the binding features of the same allele are highly correlated. In contrast, those of different alleles show low correlation. This phenomenon can be interpreted as the conservation of allele-binding properties, meaning that the key regions of alleles play a similar role across different peptide sequences. These conserved features are extracted by our deep learning model, leading to similarities among them. Notably, there is a stronger correlation among *HLA-C* features, consistent with previous studies [37,38], indicating that *HLA-C* alleles with similar evolutionary characteristics have more consistent binding preferences. To provide a global view of the learned feature space, we applied t-distributed stochastic neighbor embedding dimensionality reduction on the features extracted from the final fully connected layer on the positive data. As shown in Fig. 2E, the latent space exhibits clear boundaries partitioned by HLA family (*HLA-A*, *HLA-B*, and *HLA-C*), with partial overlap within each family. This clustering pattern confirms that BeitAI-pHLA is capable of capturing family-level binding specificities.

### Advanced accuracy and robustness on MA data

**Fig. 3.**
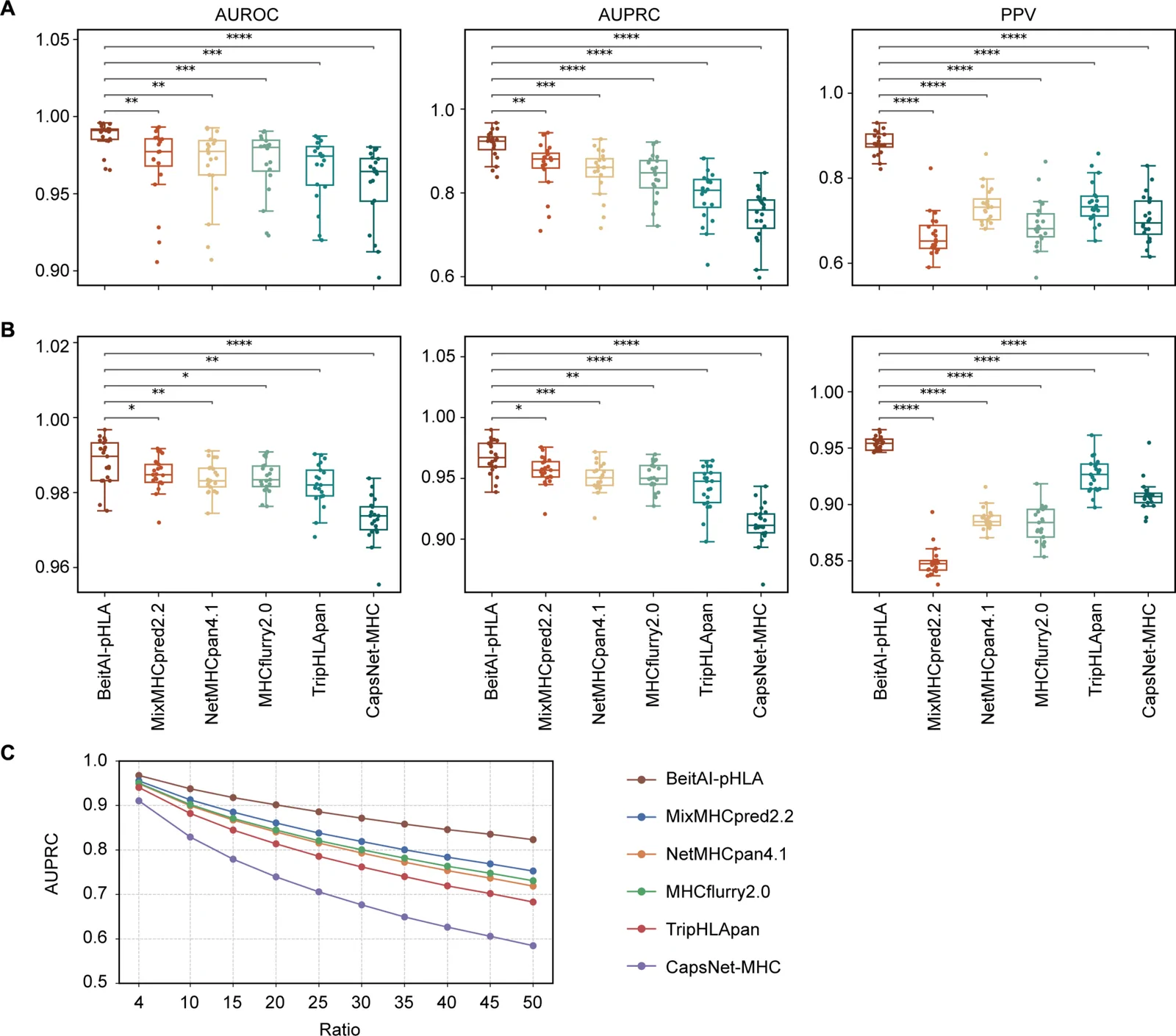
Performance comparison between BeitAI-pHLA and other advanced predictors on multiallele (MA) data. Area under the receiver operating characteristic curve (AUROC), area under the precision-recall curve (AUPRC), and positive predictive value (PPV) performance metrics on the leave-one-out dataset (A) and the MA external dataset (B). (A) The significance of differences between BeitAI-pHLA and the second-best model was assessed using the Benjamini–Hochberg procedure for multiple comparisons, yielding adjusted *P* values of 1.95 × 10^−3^, 1.95 × 10^−3^, and 1.43 × 10^−7^ for AUROC, AUPRC, and PPV, respectively. (B) The corresponding adjusted *P* values for AUROC, AUPRC, and PPV were 4.69 × 10^−2^, 1.03 × 10^−2^, and 4.27 × 10^−7^, respectively. (C) AUPRC changes under different decoy peptide ratios. The x-axis corresponds to the denominator in the decoy ratio (positive-to-negative). For instance, an x-axis value of 4 denotes a decoy ratio of 1:4, with higher values indicating a proportionally greater number of negative samples.

Immunopeptidomics experiments generate allele-specific data, a substantial fraction of which are MA data. Peptides identified in these datasets may bind to any of several alleles expressed by an individual, posing a key challenge for modeling MA data. The approach used by BeitAI-pHLA for handling MA data is detailed in Materials and Methods. We initially tested the BeitAI-pHLA predictor on the leave-one-out dataset, selecting the top 20 cell lines based on the number of entries to reduce the impact of cell lines with fewer entries. As shown in Fig. 3A, BeitAI-pHLA achieved substantially higher AUC, AUPRC, and PPV compared with other advanced predictors (MixMHCpred2.2, NetMHCpan4.1, MHCflurry2.0, TripHLApan, and CapsNet-MHC [33]). To further evaluate model performance, we tested BeitAI-pHLA on an external MA evaluation dataset. The results show that BeitAI-pHLA again achieved the best performance across metrics, with AUROC, AUPRC, and PPV values of 0.988, 0.967, and 0.954, respectively (Fig. 3B; detailed results in Table S4).

In the training data, the ratio of positive ligands to decoy peptides was approximately 1:20. In contrast, in the MA evaluation dataset, this ratio was 1:4. To explore the potential impact of different decoy peptide ratios on the results, we randomly sampled the positive data in the MA dataset while keeping the negative data constant, achieving varying decoy peptide ratios from 1:4 to 1:50 at intervals of 5. According to Fig. 3C, the performance (AUPRC) of all predictors declines as the proportion of decoy peptides increases. BeitAI-pHLA exhibited the slowest decline, while the decline rates of the other tools, ranked from the slowest to the fastest, were MixMHCpred2.2, MHCflurry2.0, NetMHCpan4.1, TripHLApan, and CapsNet-MHC. In summary, we conclude that BeitAI-pHLA demonstrates superior classification performance on MA data and retains leading performance under high decoy peptide ratios.

### The attention-based MIL mechanism refines immunopeptidomics deconvolution

**Fig. 4.**
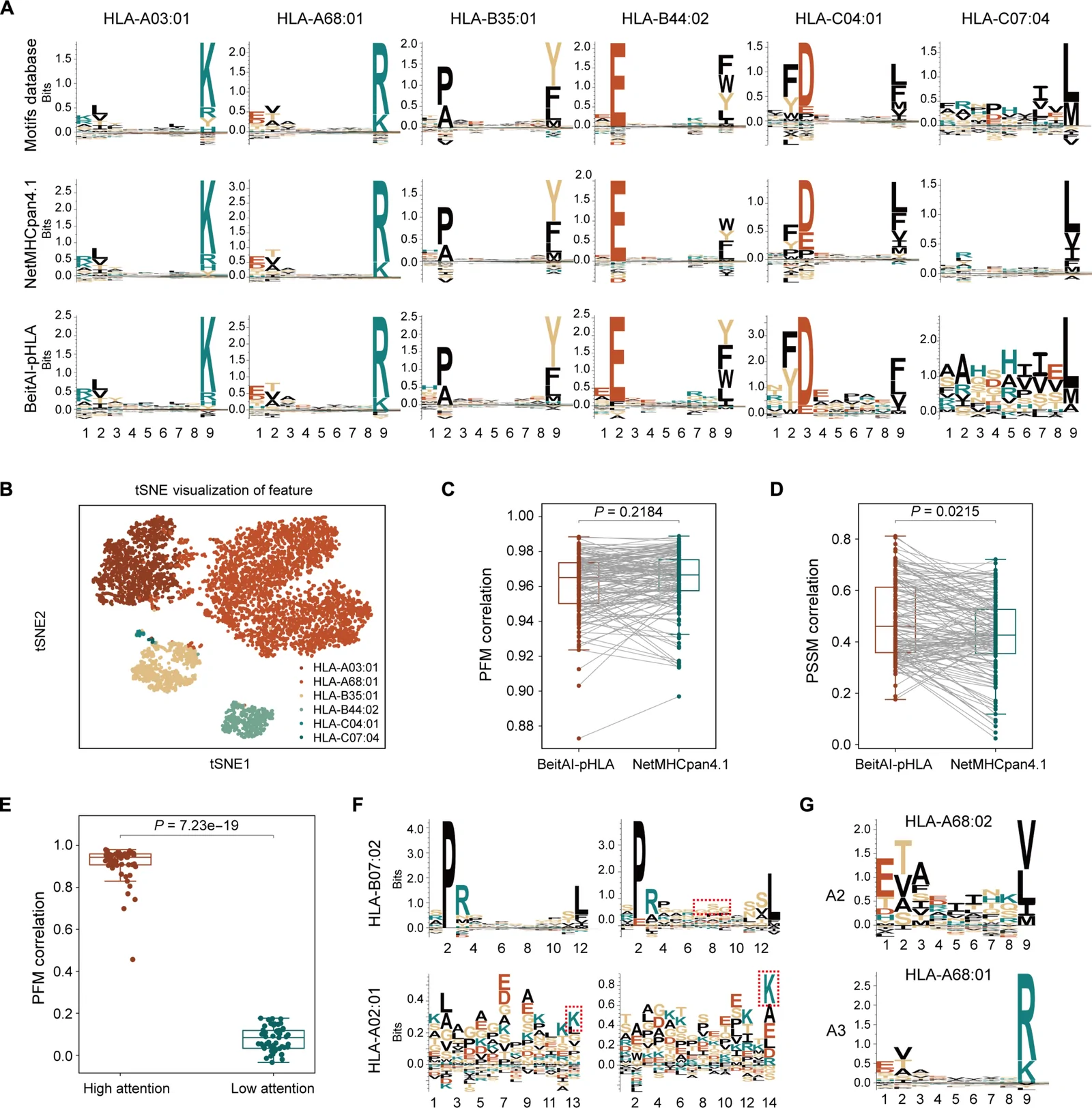
Deconvolution on multiallele data. (A) BeitAI-pHLA and NetMHCpan4.1 deconvolution motifs for the 4037-DC_I cell line. The motif comparison benchmark was derived from the MHC Motif Atlas database. (B) The binding feature representations of all positive peptides were extracted for the 4037-DC_I cell line and were visualized using t-distributed stochastic neighbor embedding (tSNE). (C) Motif similarity of BeitAI-pHLA and NetMHCpan4.1 to the benchmark motifs based on position frequency matrix (PFM). (D) Motif similarity of BeitAI-pHLA and NetMHCpan4.1 to the benchmark motifs based on position-specific scoring matrix (PSSM). Note that only motifs with more than 100 peptides were retained. (E) The correlation between the motifs from the high-attention and low-attention groups and the reference motifs from the MHC Motif Atlas. (F) BeitAI-pHLA predicted binding motifs of long peptides for human leukocyte antigen (HLA)-*B07:02* and *HLA-A02:01*. (G) Supertype differentiation between serologically similar alleles. Deconvolved motifs for *HLA-A68:01* and *HLA-A68:02* reveal distinct binding preferences.

The process of assigning peptides to homologous alleles is known as deconvolution, which represents a key challenge in modeling data containing multiple alleles. Therefore, our objective is to deconvolve MA data while learning the binding specificities of all HLA alleles. GibbsCluster [39] and MixMHCpred [25] are 2 unsupervised clustering tools for deconvolving eluted ligand MA data that have been applied in numerous studies. These deconvolution tools require either a preset or an iterative determination of the number of clusters, which may lead to discrepancies in the number of HLA-specificity clusters compared to the actual number. For instance, in scenarios involving low-expression *HLA-C* alleles, incompletely deconvoluted results may arise from overlapping binding motifs. Unlike these tools, the core algorithm NNAlign_MA [15] of NetMHCpan4.1 employs a semisupervised method to annotate MA data to single alleles without requiring manual assignment of the correct number of clusters. Similar to NNAlign_MA, BeitAI-pHLA can generate specificity peptide clusters for all alleles expressed by each cell line, with the number of clusters matching the number of HLA alleles expressed by the cell line. Using the peptide motifs published in the MHC Motif Atlas [8] as a benchmark, we constructed logo diagrams for all HLA alleles expressed by each cell line in the MA external dataset, visualizing the deconvoluted motifs. Figure 4A shows that both BeitAI-pHLA and NetMHCpan4.1 completed the deconvolution of the 6 HLA alleles expressed by the 4037-DC_I cell line, with motifs for *HLA-A* and *HLA-B* aligning with the benchmark, and motifs for *HLA-C* showing slight variations. The visualization of the allele binding feature representations extracted by the model indicates that BeitAI-pHLA can clearly distinguish several alleles (Fig. 4B). We further explored the consistency between the HLA binding motifs derived from deconvolution and those identified by single allele ligands, comparing the PCC of all allele motifs across 10 samples against the MHC motif atlas (Fig. 4C). Both BeitAI-pHLA and NetMHCpan4.1 achieve high similarity to the reference motif in terms of PFM (0.961 *vs.* 0.963, *P* = 0.2184), indicating accurate modeling of overall amino acid preferences. However, BeitAI-pHLA shows a superior correlation in the more biologically informative PSSM (0.485 *vs.* 0.427, *P* = 0.0215; Fig. 4D), representing a 13.6% improvement, which accounts for background frequencies and emphasizes energetically favorable interactions. This improvement suggests that BeitAI-pHLA not only reproduces the observed binding patterns but also better learns the underlying biophysical preferences, accurately assigning peptides to their cognate alleles within the MA scenario.

To assess whether higher attention weights were indeed assigned to alleles with the correct binding motifs, we recorded the attention scores that BeitAI-pHLA attributed to each HLA allele. Peptides were then grouped into high-attention and low-attention categories based on the top 10% and bottom 10% of attention scores, respectively. For each allele, sequence logos and motif PFM matrices were generated separately using peptides from the high- and low-attention groups, and their correlations with the reference motifs from the MHC Motif Atlas were computed. As shown in Fig. 4E, the high-attention group exhibited strong motif concordance, whereas the low-attention group showed markedly weaker motif correlation. Figure S2 further illustrates the substantial divergence in motif patterns under different attention weight assignments. These observations indicate that the attention mechanism indeed learns to prioritize alleles whose motifs align with known binding patterns. Thus, attention scores serve not merely as mathematical weights but also carry clear biological interpretability, providing a reliable basis for peptide assignment.

To further assess the biological relevance of our deconvolution results, we examined the allele-specific binding motifs inferred by BeitAI-pHLA. Classical motifs of *HLA-B07:02* are characterized by a proline at *P*_2_ and hydrophobic residues (such as leucine) at *P*_9_. Interestingly, in the MA test dataset, the 13-mer motif predicted for *HLA-B07:02* displayed a marked enrichment of glycine at central positions (e.g., positions 7, 8, and 9) (Fig. 4F and Fig. S3). This pattern suggests the presence of a noncanonical anchoring mode. In the presentation of long peptides derived from the tumor antigen NY-ESO-1 [40], *HLA-B07:02* has been reported to display similar motif features. Central glycine residues promote peptide bulging and expand the conformational space of binding, thereby reducing reliance on classical anchor interactions. Similarly, for *HLA-A02:01*, length-extended peptides (13 to 14 amino acids) showed unexpected enrichment of lysine at the C terminus (Fig. 4F and Fig. S3), while the PΩ pocket of *A02:01* usually favors hydrophobic residues. Structural analyses have shown that terminal charged residues can induce conformational adjustments [41], such as the repositioning of Tyr-84 or the lifting of Lys-146, that open the F pocket and allow C-terminal extension toward solvent, thereby broadening TCR recognition.

We also examined how the deconvolved motifs inferred by BeitAI-pHLA relate to established HLA supertypes. For instance, the A2 supertype is characterized by aliphatic or hydrophobic residues at *P*_2_ and *P*Ω. In contrast, the A3 supertype preferentially binds small or aliphatic residues at *P*_2_ and basic residues (R and K) at the C terminus. Notably, although *HLA-A68:01* and *HLA-A68:02* fall within the same serological group (*HLA-A68*), they belong to different supertypes (Fig. 4G). Structural studies have shown that *HLA-A68:02* possesses a C-terminal pocket resembling that of *HLA-A02:01*, with a hydrophobic Tyr forming the base, whereas *HLA-A68:01* features a negatively charged Asp in this position, favoring basic C-terminal anchors [42]. These local structural differences, particularly in the F-pocket electrostatics, can shift peptide preferences despite overall structural similarity and contribute to functional complementarity across supertypes, thus broadening pathogen peptide coverage at the population level.

To assess the reliability of our hard-assignment deconvolution, we analyzed the difference between the highest and second-highest attention scores for each peptide in the MA external dataset. Analysis of attention score differences (Fig. S4) shows that ~5% of assignments show near-equal attention scores between 2 alleles (Δ ≤ 0.1). This analysis confirms that the vast majority of peptides are unambiguously assigned to a single dominant allele. These findings demonstrate that BeitAI-pHLA not only achieves accurate deconvolution but also captures the structural and biochemical principles underlying HLA-I binding specificity, reinforcing the biological validity of its attention-based MIL inference.

### Validation on immunogenic neoepitopes

**Fig. 5.**
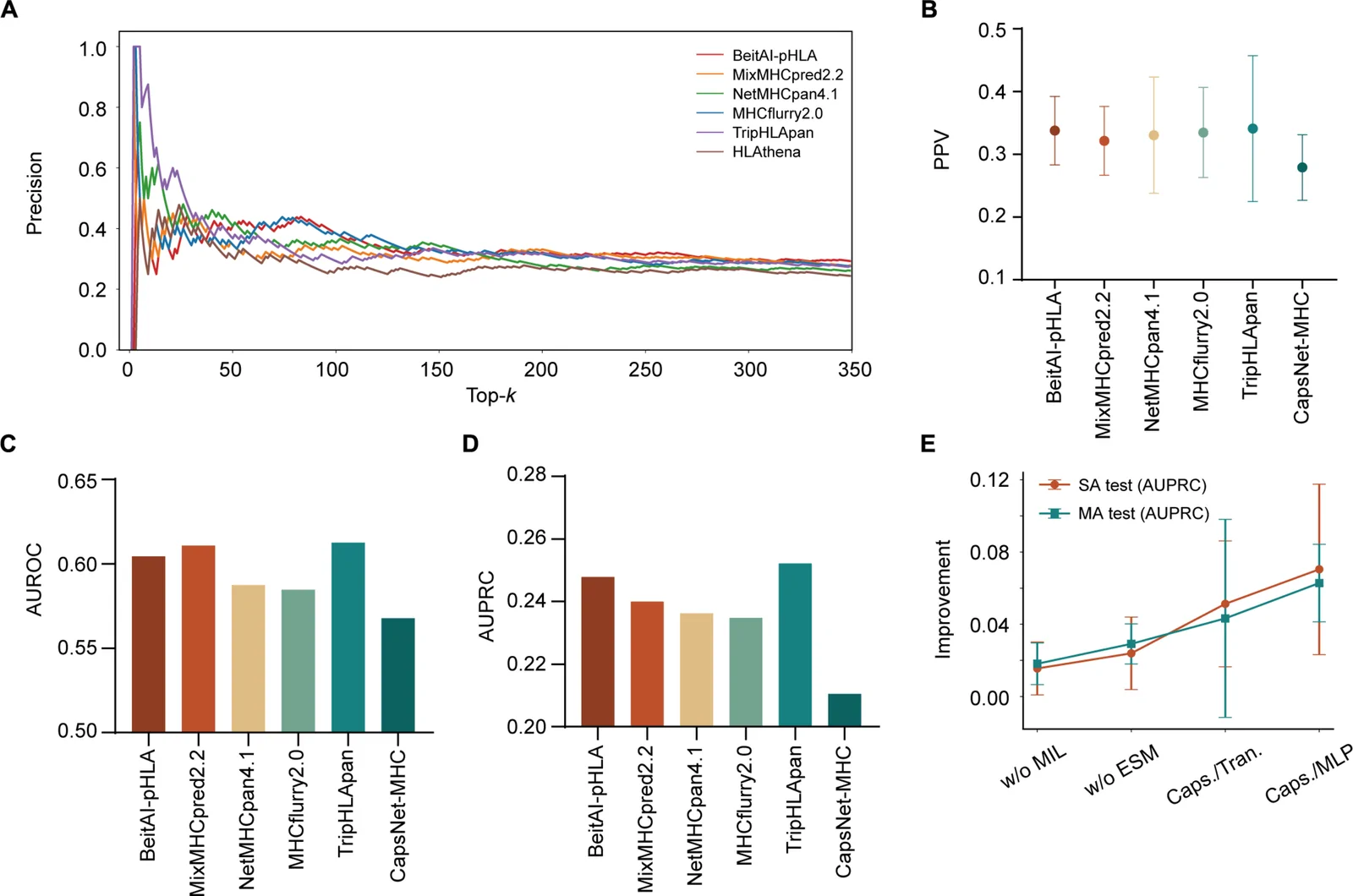
Model performance on immunogenic neoepitopes evaluation dataset and Ablation analysis. (A) Precision of top-ranked immunogenic neoepitopes. (B)The average positive predictive value (PPV) of BeitAI-pHLA over all top-*k* ranked candidate neoepitopes. (C) Area under the receiver operating characteristic curve (AUROC) and (D) Area under the precision-recall curve (AUPRC) performance metrics on the immunogenic neoepitopes evaluation dataset. (E) Comparison among BeitAI-pHLA without multi-instance learning (MIL) (w/o MIL), BeitAI-pHLA without ESM (w/o ESM), BeitAI-pHLA with the capsule module replaced by a Transformer (Caps./Tran.), and BeitAI-pHLA with the capsule module replaced by a multilayer perceptron (MLP) (Caps./MLP).

Immunogenic neoepitopes are valuable targets for cell therapy and peptide-based vaccines. Accurate prediction of peptide binding to HLA class I molecules can effectively identify candidates for immunogenic neoepitopes. Nevertheless, most candidates lack immunogenic properties, and experimental methods to validate their immunogenicity are low-throughput. Despite these challenges, HLA presentation remains a crucial step in eliciting an immunogenic response. We used the immunogenicity evaluation dataset to evaluate the model’s ability to predict these immunogenic epitopes and compared it with 5 peer tools (see Table S5 for the raw prediction results). In neoantigen prediction, typically only a few top-ranked candidates are selected for clinical validation. We calculated the precision of the top-*k* ranked peptides (Precision@K, Fig. 5A) and their overall PPV (*k* = 1 to 349, Fig. 5B). The combined analyses show that BeitAI-pHLA ranked among the top 2 at most *k* values, and its average PPV was comparable to that of other advanced tools. To further evaluate performance across the entire dataset, we calculated the AUROC (Fig. 5C) and AUPRC (Fig. 5D). BeitAI-pHLA achieved the second-best AUPRC (0.248), exceeding TripHLApan (0.252), MixMHCpred2.2 (0.240), NetMHCpan4.1 (0.236), MHCflurry2.0 (0.235), and CapsNet-MHC (0.210). These results demonstrate that BeitAI-pHLA can provide a higher-quality shortlist for downstream experimental validation, thereby improving the efficiency of identifying immunogenic neoepitopes.

### Ablation analysis

To evaluate the contributions of each module of BeitAI-pHLA to the prediction performance, we conducted ablation studies. We focused on the impact of 3 key modules: the ESM protein language model, the capsule neural network module, and the MIL module. To further clarify the contribution of the capsule network module, we introduced 2 additional baseline models (a Transformer encoder and an MLP, with parameters detailed in Materials and Methods) to replace the capsule layer. As shown in Fig. 5E, all modules contributed to improved prediction performance, with the capsule network module providing the greatest gain. The capsule-based architecture consistently outperformed both baseline models across different datasets (on the SA and MA datasets, its AUPRC improved by 0.016 to 0.070 over the Transformer and MLP models, respectively). This analysis confirms that the capsule network is more suitable than Transformer or fully connected (dense) architectures for capturing the hierarchical and spatial features that are critical for peptide–HLA binding prediction.

## Discussion

With advances in MS technology, immunopeptidomics data are rapidly accumulating. Large-scale open-source MS peptidomics datasets offer great promise for applying deep learning to predict HLA ligand presentation. In recent years, tools built on protein language models and transformer architectures (e.g., TransHLA) have substantially advanced HLA–peptide binding prediction. However, these methods are primarily optimized for affinity prediction or single-allele settings and lack dedicated solutions for the inherent multispecificity challenge in MA immunopeptidomics. Against this background, we propose BeitAI-pHLA, a deep learning model designed to predict the presentation of antigen sequences by HLA class I molecules. Unlike peer methods such as NetMHCpan and MHCflurry, BeitAI-pHLA uses a protein language model pretraining approach to encode peptide and HLA sequences, rather than a static BLOSUM matrix. This amino acid embedding method leverages transfer learning features to enable high-dimensional representation and enhance adaptability to novel datasets. Second, we have integrated deep neural networks and capsule networks to effectively capture binding features. To address the multispecificity of MA data, BeitAI-pHLA directly models on MA data, dynamically allocating alleles through an attention mechanism. This batch-granularity update strategy enables more thorough feature learning compared with NNAlign_MA’s epoch-granularity approach.

The combination of these factors results in BeitAI-pHLA demonstrating outstanding performance, particularly on MA data. Specifically, we evaluated BeitAI-pHLA along with 5 other advanced prediction tools across multiple datasets. Evaluation results indicate that BeitAI-pHLA significantly outperforms other tools on MA datasets, with an AUPRC of 0.967 attained on the external validation dataset. Compared with the existing deconvolution method, NetMHCpan4.1, the motifs discovered show higher motif similarity to benchmarks in HLA motif maps (13.6% improvement). These results suggest that BeitAI-pHLA effectively captures the binding motifs of HLA class I alleles, owing to its attention-based MIL mechanism. This mechanism enables the model to identify alleles whose motifs align with known binding patterns and assign them higher weights, thereby determining the allele restriction of HLA-I ligands. Furthermore, we evaluated the model’s performance on an independent neoepitope dataset to assess its ability to predict immunogenic neoepitopes. Although immunogenicity is a multifactorial process involving proteasomal cleavage, TAP transport, TCR recognition, and other intracellular events, HLA binding remains a key determinant. Compared with other tools, BeitAI-pHLA achieved the second-best AUPRC on the immunogenic neoepitope dataset.

Despite the promising performance of our method across multiple evaluation datasets, several limitations should be noted. First, potential contaminants in immunopeptidomics data, such as residual peptides from other samples, substances that do not bind to HLA molecules, and incorrectly annotated peptides, constitute noise. This noise can be misassigned to different alleles during deconvolution, leading to biases in binding motif assignments. To enhance the robustness of neoantigen prediction, BeitAI-pHLA’s presentation probability can be integrated with multiomics data. By combining it with RNA expression levels and mutation allele frequencies from source genes, we can prioritize high-expression, biologically relevant candidate neoantigens. Furthermore, integrating TCR sequencing data enables validation of whether the presented peptides trigger T-cell clonal expansion in vivo. This multidimensional cross-validation strategy helps mitigate noise from single data sources, significantly improving the reliability of clinical target screening. Second, we observed low prediction accuracy in several tools (including BeitAI-pHLA) for immunogenic epitope prediction and found that only a small fraction of predicted positive peptides are truly immunogenic. Similar to other mainstream tools, BeitAI-pHLA focuses primarily on the final step of the antigen presentation pathway: pHLA binding. BeitAI-pHLA can therefore complement tools that specialize in earlier stages of antigen processing, such as NetChop for proteasomal cleavage prediction [43] and NetCTL for TAP transport prediction [44]. An ideal future direction is to integrate the accurate binding-prediction capability of BeitAI-pHLA with these antigen-processing models, thereby enabling a more comprehensive end-to-end pipeline that spans from raw protein sequences to the final identification of immunogenic neoepitopes. Moreover, the current version of our algorithm concatenates peptide and HLA sequences and does not incorporate 3-dimensional structural features of pHLA interactions, thereby missing important biophysical determinants of binding. Future improvements may include integrating cross-attention mechanisms or Evoformer-style interaction layers, as well as developing hybrid strategies that combine sequence-based deep learning with physics-based energy scoring. These enhancements have the potential to bridge this limitation.

In summary, our work presents a novel and general tool for HLA-I epitope prediction and immunopeptidomics analysis, significantly improving antigen presentation prediction performance. In the development of personalized immunotherapy, BeitAI-pHLA can serve as a key computational module integrated directly into the neoantigen discovery pipeline. By utilizing tumor genomic data from patients, the tool performs high-precision HLA-I presentation predictions on mutated peptides, rapidly identifying high-quality candidate neoantigens. These predictions can then be further integrated with multiomics data, such as transcriptomics, to significantly enhance the success rate of subsequent experimental validations. This efficient approach provides unique opportunities for personalized cancer treatment and tumor vaccine development. In the future, high-quality MS and immunogenicity training data, along with more advanced deep learning frameworks (e.g., partial fine-tuning of protein language models and structure-aware architectures), will further refine neoepitope identification tasks.

## Conclusions

In this study, we developed BeitAI-pHLA, a new deep learning framework that integrates a protein language model (ESM), capsule neural networks, and an attention-based MIL mechanism for accurate pHLA class I binding prediction. By directly modeling MA data and dynamically assigning peptides to their cognate alleles, our approach addresses a key limitation in immunopeptidomics analysis. BeitAI-pHLA demonstrated high accuracy in MA scenarios and maintained robust performance across varying decoy-peptide ratios. Furthermore, BeitAI-pHLA exhibits enhanced motif deconvolution capability and shows the second-best AUPRC for immunogenic neoepitope prediction. This work provides a powerful and reliable computational tool for immunopeptidomics analysis, with potential applications in targeted immunotherapy and vaccine development. Future efforts will focus on incorporating additional biological context to enhance prediction accuracy further.

## Data Availability

The MS ligand dataset is available at https://services.healthtech.dtu.dk/suppl/immunology/NAR_NetMHCpan_NetMHCIIpan/. The remaining datasets (including results on the MA evaluation datasets) and the source code are available at https://github.com/KindstarGlobalInstitute/BeitAI-pHLA. BeitAI-pHLA webtool is freely accessible for academic use via its online web server at https://phla.kindstarbiotech.com.
