# Supplementary material for: BeitAI-pHLA: Multiallele Peptide–HLA Class I Binding Prediction Using Protein Language Model and Multi-Instance Learning
Source: Comput Struct Biotechnol J. 2026 Sep 11;35(1):0220. doi: 10.34133/csbj.0220 (PMC13562803; doi:10.34133/csbj.0220)
Supplement: Supplementary file 1 [file csbj.0220.f1.zip › Supplementary.docx]

## Capsule neural network architecture (formula derivation)

In this section, we provide the detailed mathematical formulation for the capsule network operations described in the main text.

The inputs $u_{i}$ from the convolutional layer are passed to the higher-level capsules via a transformation weight matrix $W_{ij}$, as described by the following equation:

$$\hat{u}_{j|i}=W_{ij}\cdot u_{i}$$

This process captures both local features and their relative positions. The features are then aggregated using weight vectors $c_{ij}$ through dynamic routing, where:

$$s_{j}=\sum_{i} c_{ij}\hat{u}_{j|i}$$

The attention weights $c_{ij}$ are calculated using the softmax function:

$$c_{ij}=softmax\left( b_{ij} \right)$$

where $b_{ij}$ serves as an intermediate variable that is updated as follows:

$$b_{ij}=b_{ij}+v_{j}\hat{u}_{j|i}$$

Finally, the vector $v_{j}$ is updated using the squash function:

$$v_{j}=\frac{\left\| s_{j} \right\|^{2}}{1+\left\| s_{j} \right\|^{2}}\cdot\frac{s_{j}}{\left\| s_{j} \right\|}$$

This formula resembles the activation functions used in conventional neural networks, enabling capsules to represent both local and global feature information effectively.

## Multi-instance learning architecture (formula derivation)

In our framework, each multi‑allele sample is treated as a **bag** $B=\{x_{1},x_{2},\ldots,x_{m}\}$, where each $x_{1}\in\mathbb{R}^{200}$ is the capsule output for the *i*-th HLA allele expressed by that sample, and *m* varies across samples (typically 2–6 for human cell lines).

For each attention head $h\in\{1,2,3\}$, we compute a normalized attention score for each instance:

In the attention mechanism, the attention scores are computed as follows:

$${Attention}_{i}^{(h)}=softmax\left( V_{i}^{(h)}W^{(h)} \right)$$

where $V_{i}^{(h)}$is the transformed representation of instance 𝑖, and $W^{(h)}$ is a learnable weight matrix for head *h*. The final multi‑head attention is obtained by concatenating the outputs of all three heads:

$$Attention=Concat\left( {Attention}^{(1)}, {Attention}^{(2)}, {Attention}^{(3)} \right) .$$

Equivalently, for each instance *i*, the aggregated attention weight across heads is:

$$\alpha_{i}=\frac{1}{3}\sum_{h=1}^{3} \frac{exp(\boldsymbol{w}_{h}^{T}\tanh(\boldsymbol{V}_{h}x_{i}))}{\sum_{j=1}^{m} exp(\boldsymbol{w}_{h}^{T}\tanh(\boldsymbol{V}_{h}x_{i}))}$$

The final bag‑level representation is:

$$z=\sum_{i=1}^{m} \alpha_{i}x_{i}$$

which is fixed‑dimensional regardless of *m*, enabling the model to handle variable numbers of HLA alleles per sample. The capsule layer (20 capsules × 10‑dim vectors) serves as a feature extractor capturing diverse binding motifs; the MIL attention then operates on these allele‑specific features to resolve multi‑allele ambiguity. The computed attention scores are then fed into a fully‑connected block followed by a sigmoid output layer to produce the final binding prediction.

## Model parameter counts

BeitAI‑pHLA comprises a frozen ESM2‑150M backbone (150M parameters) and ~12.4M trainable parameters. For comparison: NetMHCpan4.1 (~0.5M), MHCflurry2.0 (~1.2M), MixMHCpred2.2 (~0.2M), TripHLApan (~2.5M), and CapsNet‑MHC (~2.8M). Despite a larger trainable parameter count than most tools, the frozen ESM backbone adds no training overhead, and the performance gains justify this design.

## Supplementary Figure 1

**Figure S1. Residue importance for a canonical epitope.** The amino acid substitution heatmap for the influenza epitope GILGFVFTL (HLA-A*02:01) shows P2 (I) and P9 (L) as the primary anchors, consistent with known binding motifs.

## Supplementary Figure 2

**Figure S2. Allele motif logo plots based on different attention score distributions.** The sequence logo plot for HLA-B07:02 shows that high-attention peptides (upper panel) reproduce the typical P2-proline/PΩ-leucine motif, while low-attention peptides (lower panel) exhibit incorrect residue preferences. A similar misassignment is observed in HLA-B15:01.

## Supplementary Figure 3

**Figure S3. BeitAI-pHLA predicted binding motifs for HLA-B07:02 and HLA-A02:01 in peptides of lengths 11-14.** For HLA-B07:02, glycine is enriched at the internal positions (P7-P9) of the 13-mer peptide. For HLA-A02:01, a C-terminal lysine preference is observed in the 12-mer peptide.

## Supplementary Figure 4

**Figure S4. Distribution of attention score differences for multi‑allele assignments.** Histogram of Δ = Top1 − Top2 attention scores across all peptides in the multi‑allele external dataset.

## Supplementary Table S1

**Table R3.** The edit distances between each held‑out allele and its closest training allele, based on the 34‑residue HLA pseudo‑sequence.

| **SA test allele** | **Rows** | **Edit distance (of 34)** | **No. training alleles with d ≤ 4** | **No. training alleles with d ≤ 5** | **Cluster size** |
| --- | --- | --- | --- | --- | --- |
| HLA-A01:01 | 84,823 | 9 | 0 | 0 | 2 |
| HLA-A01:03 | 82 | 10 | 0 | 0 | 2 |
| HLA-A32:01 | 13,676 | 6 | 0 | 0 | 1 |
| HLA-B07:02 | 201,038 | 4 | 1 | 1 | 1 |
| HLA-B15:42 | 30 | 5 | 0 | 1 | 1 |
| HLA-B37:01 | 400 | 7 | 0 | 0 | 1 |
| HLA-B47:01 | 366 | 7 | 0 | 0 | 1 |
| HLA-B73:01 | 3,293 | 8 | 0 | 0 | 1 |
| HLA-C17:01 | 8,587 | 6 | 0 | 0 | 1 |
| Total | 312,295 | median 7, mean 6.9 |  |  |  |

## Supplementary Table S2

**Table S2.** Performance of two HLA input strategies (full-length and pseudo sequence) on the SA and MA datasets.

| **HLA Input** | **Dataset** | **AUPRC** | **AUROC** | **F1** | **Pre.** | **Rec.** |
| --- | --- | --- | --- | --- | --- | --- |
| Pseudo‑sequence | SA | 0.7894 | 0.9337 | 0.7626 | 0.8466 | 0.6937 |
| Full‑length | SA | 0.7870 | 0.9313 | 0.7631 | 0.7917 | 0.7365 |
| Pseudo‑sequence | MA | 0.8872 | 0.9815 | 0.8279 | 0.8945 | 0.7705 |
| Full‑length | MA | 0.8801 | 0.9785 | 0.8286 | 0.8320 | 0.8251 |
